# Bibliometric mapping of scientific production on photodynamic therapy in cutaneous wounds: A web of science analysis

**DOI:** 10.1007/s10103-026-04987-1

**Published:** 2026-08-11

**Authors:** Victor Barros Fracalossi, Camila Maria Ribeiro Pacheco, Elaine Caldeira de Oliveira Guirro

**Affiliations:** Ribeirão Preto Medical School, Department of Health Sciences, University of São Paulo, São Paulo, Brazil

**Keywords:** Bibliometrics, Photodynamic therapy, Cutaneous wounds, Wound healing, Science mapping

## Abstract

Cutaneous wounds, particularly chronic wounds, represent a major clinical and public health challenge because of their high prevalence, prolonged healing time, and substantial healthcare and economic burden. In this context, photodynamic therapy (PDT) has attracted growing interest owing to its antimicrobial potential and ability to modulate biological processes involved in tissue repair. This bibliometric study used the Web of Science Core Collection. Articles published between 2000 and 2025 were included without language restrictions. Bibliographic records were analyzed using VOSviewer and CitNetExplorer to characterize temporal trends in scientific production and the collaborative, conceptual, and intellectual structure of the literature. A total of 1,008 articles were identified. Scientific production increased markedly, particularly from 2020 onward, accompanied by the consolidation of international collaboration networks and the predominance of China, the United States, and Brazil among the most productive countries. Bibliometric analyses revealed a progressive reconfiguration of the field’s conceptual structure. Initially centered on the microbiological control of wounds, the literature later incorporated biomaterials, nanotechnology, smart hydrogels, and multifunctional therapeutic platforms aimed at tissue repair. Historiographic analysis showed that this evolution occurred cumulatively, preserving the foundations established by pioneering studies while progressively incorporating newer biological and technological approaches. Research on PDT for cutaneous wounds is expanding and becoming increasingly interdisciplinary. The integration of photomedicine, nanotechnology, biomaterials science, and regenerative medicine appears central to the field’s recent reorganization, providing a basis for future research and for the development of innovative therapeutic strategies for cutaneous wound management.

**Supplementary Information:**

The online version contains supplementary material available at 10.1007/s10103-026-04987-1.

## Introduction

Cutaneous wounds comprise a heterogeneous group of injuries characterized by disruption of skin integrity and may result from diverse etiologies, including trauma, burns, surgical procedures, vascular, metabolic, or infectious diseases [1]. Among these injuries, chronic wounds constitute an important clinical and public health challenge. They are defined as cutaneous wounds that fail to progress through the orderly and predictable phases of healing and remain unclosed within 12 weeks [2, 3].

The etiology of chronic wounds is multifactorial and commonly involves complications of diabetes mellitus, venous insufficiency, inadequate arterial perfusion, and prolonged immobility [4]. Beyond compromised tissue integrity, these lesions adversely affect patients’ functioning, independence, and quality of life, causing persistent pain, physical limitations, psychological distress, and substantial healthcare costs [5–8].

Cutaneous wound healing involves coordinated events encompassing the phases of hemostasis, inflammation, proliferation, and remodeling [9, 10]. In chronic wounds, these phases may be interrupted or stalled, particularly during the inflammatory phase, resulting in a dysregulated tissue microenvironment characterized by excessive pro-inflammatory cytokines, extracellular matrix degradation, and reduced growth factor levels [11–13]. Persistent infection and microbial biofilm formation constitute additional barriers, hindering bacterial burden control and impairing the regenerative response [14, 15]. In this context, therapeutic approaches capable of modulating inflammation, effectively controlling resistant microorganisms and microbial biofilms, and promoting healing are essential for the management of chronic cutaneous wounds.

Among these approaches, photodynamic therapy (PDT) has emerged as a promising therapeutic alternative for the treatment of cutaneous wounds, particularly chronic and hard-to-heal wounds [16, 17]. PDT is based on the interaction between a photosensitizing agent, a light source at a specific wavelength, and molecular oxygen, which promotes photochemical reactions that generate reactive oxygen species capable of damaging microbial structures [18, 19]. Experimental and clinical studies suggest that PDT may exert antimicrobial, anti-inflammatory, pro-angiogenic, and biostimulatory effects, contributing to infection control and tissue repair [20–22]. Beyond conventional antimicrobial applications, recent advances have explored nanostructured delivery systems, photosensitive hydrogels, and smart formulations that may enhance the therapeutic efficacy of PDT in different wound-healing contexts.

Over recent decades, the literature on PDT applied to cutaneous wounds has expanded considerably, encompassing in vitro studies, animal models, microbiological investigations, and preliminary clinical trials [1, 23–26]. Although these studies suggest promising outcomes, important limitations persist, including small sample sizes, methodological heterogeneity, and a lack of standardization in irradiation parameters and photosensitizer concentrations. Concurrently, bibliometric analyses have been conducted in several areas of PDT, including oncology, dermatology, antimicrobial applications, and nanotechnology, contributing to the understanding of the scientific evolution of these fields. However, studies that systematically investigate the evolution of scientific production on PDT applied to cutaneous wounds remain scarce. This gap is particularly relevant because wound-healing research simultaneously involves tissue repair, biofilm control, microbial resistance, biomaterials, and translational strategies directed toward clinical practice. Recent developments reflect the growing integration of nanotechnology, smart biomaterials, and antimicrobial strategies capable of overcoming bacterial resistance mechanisms and improving biofilm management, which are key challenges in contemporary wound-healing research [27, 28]. Accordingly, the absence of specific bibliometric analyses limits the understanding of the field’s intellectual structure and hinders the identification of research trends, scientific collaboration networks, leading contributors, and knowledge gaps that may guide future investigations.

Bibliometric analysis provides a relevant methodological approach for investigating the structure and dynamics of scientific production within a given field of knowledge. By assessing indicators of productivity, citation performance, and scientific collaboration, this approach enables the identification of the main authors, journals, and countries that shape the literature, as well as the understanding of temporal evolution and predominant thematic foci. It also supports the identification of emerging areas, knowledge gaps, and patterns of scientific development that may inform future research agendas.

Therefore, the present study aimed to map and analyze global scientific production on the application of photodynamic therapy to cutaneous wounds through a bibliometric approach based on the Web of Science. Specifically, the study sought to identify temporal publication trends, leading journals, authors, countries, and scientific collaboration networks, as well as to characterize the thematic and intellectual structure of the field, contributing to a better understanding of its evolution and the identification of future development opportunities.

## Methods

This study is a bibliometric analysis of the scientific literature on photodynamic therapy applied to cutaneous wound healing, conducted to characterize the scientific evolution of the field. The analysis integrated performance analysis and science mapping techniques.

The bibliographic search was conducted in the Web of Science Core Collection (WoS), selected for its broad coverage of scientific journals, standardized bibliographic records, availability of citation data, and compatibility with specialized bibliometric analysis software, all of which support robust and reproducible analyses.

Based on the objective of this study and the bibliometric approach adopted, the following research questions were established:

- (RQ1) How has scientific production on PDT applied to cutaneous wounds evolved over time?
- (RQ2) Which journals, authors, and countries are the main contributors to scientific production on the topic?
- (RQ3) What patterns of international scientific collaboration characterize this field?
- (RQ4) Which thematic clusters, research trends, and knowledge gaps can be identified through bibliometric analysis of the literature?

The search strategy was developed in the Topic (TS) field of WoS, which searches titles, abstracts, author keywords, and Keywords Plus. Search terms were selected from descriptors commonly used in the PDT and wound-healing literature and were combined with Boolean operators to define the thematic scope. To improve specificity and minimize retrieval of records outside the scope of this study, exclusion terms were incorporated through the Boolean operator NOT. The complete search strategy was:

TS = ((“photodynamic therapy” OR “photochemotherapy” OR PDT) AND (wound* OR injury* OR “chronic wound” OR ulcer* OR “foot ulcer” OR “skin ulcer” OR “leg ulcer” OR “varicose ulcer” OR “diabetic foot ulcer” OR “pressure ulcer” OR “bed sore” OR “decubitus ulcer” OR “cutaneous wound” OR “burn wound” OR “burn wounds”) NOT (oncology OR cancer OR tumor* OR carcinoma OR melanoma OR neoplasm* OR dental OR dentistry OR periodontal OR endodontic OR wastewater OR water-treatment OR environmental OR agricultural OR plant* OR marine OR aquatic OR algae OR fungal OR yeast* OR acne OR “acne vulgaris”)).

The search was conducted on December 15, 2025. Subsequently, the filters available within the Web of Science were applied, restricting the selection to documents classified as Articles, with no restrictions regarding the language or country of origin of the publications. Given the objective of analysing the contemporary development of the field, studies published between 2000 and 2025 were included. The bibliometric corpus comprised the records retrieved after application of the search strategy and the refinement criteria established in the database.

Bibliographic records were exported in Plain Text format, including full bibliographic information and cited references, and were subsequently processed using VOSviewer version 1.6.20 (Centre for Science and Technology Studies [CWTS], Leiden University, the Netherlands) and CitNetExplorer version 1.0.0 (CWTS, Leiden University, the Netherlands). These software packages were used to construct, visualise, and analyse bibliometric networks.

Initially, descriptive bibliometric indicators were calculated concerning the temporal evolution of publications, Web of Science categories, most productive journals, authors with the highest number of publications, countries with the greatest scientific contribution, and the distribution of keywords used in the literature.

Science mapping analyses were conducted in VOSviewer using the full counting method. The collaborative structure of the literature was investigated through co-authorship networks among countries (Co-authorship – Countries) and authors (Co-authorship – Authors). A minimum threshold of five documents per unit of analysis was adopted, and no minimum citation threshold was applied to the country-level analysis. The conceptual structure was investigated through keyword co-occurrence analysis (Co-occurrence – All Keywords), considering a minimum occurrence frequency of five. For all networks, Network Visualization was generated to represent the structure of relationships among elements; Overlay Visualization was used to identify the temporal evolution of research topics based on the average publication year of the items; and Density Visualization was used to represent the concentration and intensity of bibliometric relationships.

The intellectual structure of the literature was investigated using CitNetExplorer. During data import, the option “Include non-matching cited references” was enabled, allowing the inclusion of references cited by articles in the original corpus that had not been directly retrieved by the search strategy. This procedure enabled the incorporation of cited references that play a structuring role in the historical development of the scientific field because they are widely cited by the analysed articles. Core Publications analyses were conducted to identify the central publications in the network, whereas Citation Score analyses were performed to assess the influence of publications within the citation structure.

Thematic clusters were identified using the clustering algorithm implemented in CitNetExplorer, with a resolution of 1.0, a minimum cluster size of 10 publications, a random starting point, 10 optimisation iterations, and a random seed of zero. Cluster interpretation was based on an integrated analysis of publications with the highest centrality and Citation Score, combined with the keyword co-occurrence and temporal evolution analyses obtained in VOSviewer.

Based on these analyses, graphs, tables, and bibliometric maps were developed to characterise the productive, collaborative, conceptual, and intellectual structures of the literature, as well as its dynamics and temporal evolution regarding scientific production on photodynamic therapy for cutaneous wound healing.

## Results and discussion

### Characterization of the bibliometric corpus

The search strategy retrieved a bibliometric corpus comprising 1,008 scientific articles published between 2000 and 2025, which served as the basis for all analyses performed in this study. These records were used to characterize the evolution of scientific production, the collaborative structure, the conceptual organization, and the intellectual structure of the literature on photodynamic therapy for cutaneous wound healing.

For the historiographic analysis conducted using CitNetExplorer, cited references that were not retrieved by the search strategy were automatically incorporated into the citation network, expanding it to 1,067 interconnected publications. This procedure enabled the reconstruction of the historical development of the field based on its foundational publications while preserving the distinction between publications included in the bibliometric corpus and external references incorporated exclusively into the citation network.

## Scientific evolution of the field

**Fig. 1:**
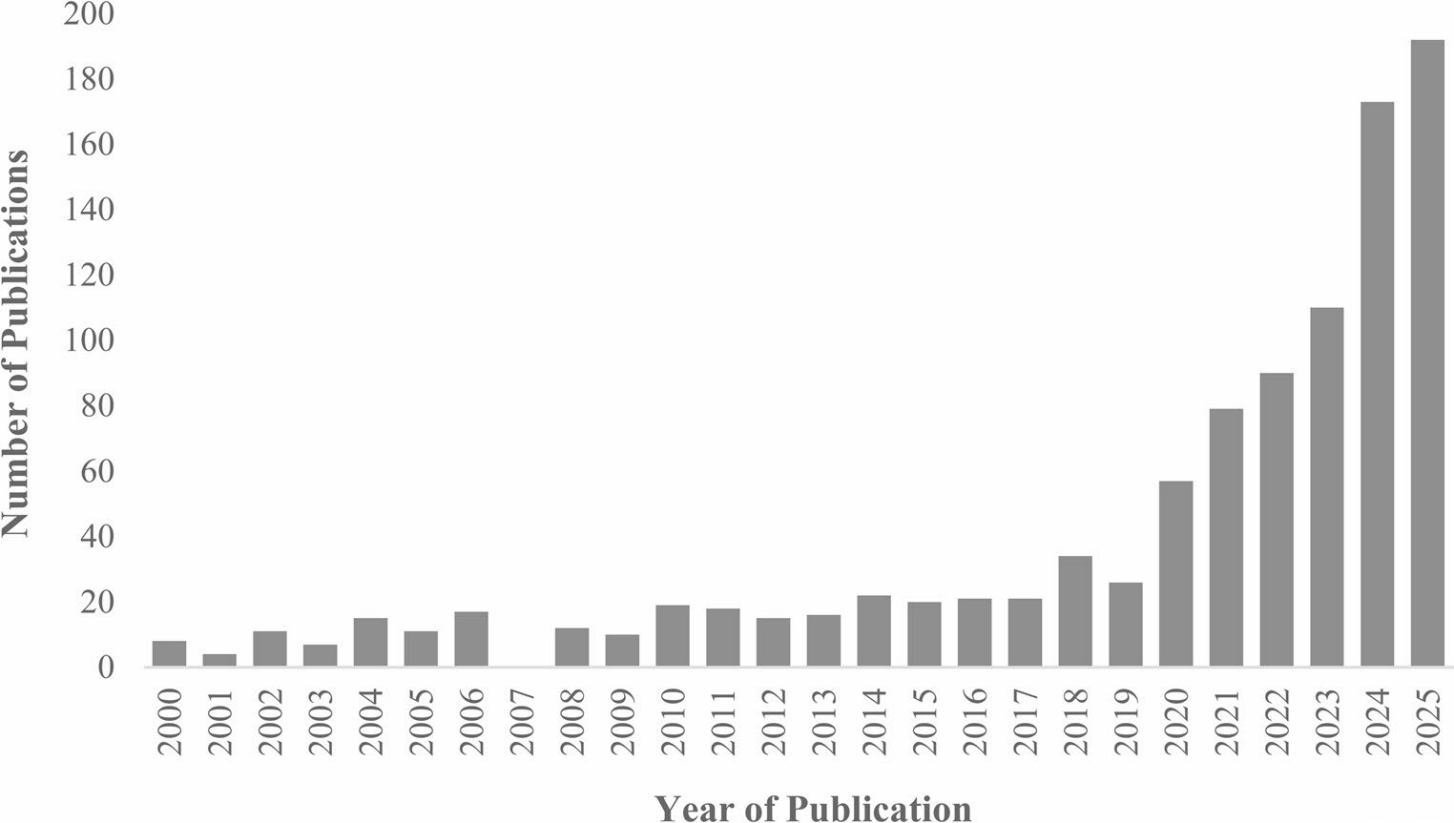
Temporal evolution of scientific production (2000–2025)

Publications were identified in nearly every year between 2000 and 2025, with the exception of 2007, for which no studies meeting the adopted search strategy were retrieved (Fig. 1). Between 2000 and 2019, the annual number of publications showed only slight fluctuations, indicating relatively stable scientific output. From 2020 onward, a marked inflection in the growth trend was observed, followed by a continuous and substantial increase in scientific production through 2025, when the highest number of publications during the study period was recorded.

From a bibliometric perspective, this temporal pattern characterizes a recent phase of expansion in the scientific field. In emerging research areas, prolonged periods of moderate growth are often followed by a phase of rapid acceleration, driven by increasing interest from the scientific community, the diversification of research lines, and the incorporation of new research groups. However, the increase in the number of publications should not be interpreted, in isolation, as an indicator of scientific maturity or clinical consolidation, since bibliometric indicators describe patterns of scientific production but do not replace methodological or clinical assessments of the included studies [29]. In this context, the observed temporal evolution indicates the recent dynamism of the field, whose comprehensive understanding depends on an integrated analysis of the productive structure, collaborative networks, and conceptual evolution of the literature, as discussed in the following sections.

## Scientific structure of the field

The expansion of scientific production was accompanied by a reorganization of the field’s scientific structure. The distribution of Web of Science categories indicates that the recent development of photodynamic therapy for cutaneous wound healing has been predominantly driven by research areas related to nanotechnology, materials science, multidisciplinary chemistry, and biomaterials (Table 1). This pattern is further supported by the profile of the most productive journals, which are predominantly dedicated to the development of advanced materials and biomedical technologies, including Chemical Engineering Journal, ACS Applied Materials & Interfaces, and Advanced Healthcare Materials, as well as the specialized journal Photodiagnosis and Photodynamic Therapy (Table 2).

**Table 1:** Top 10 web of science categories among the retrieved articles

Web of science category	Number of articles	% of Total
Nanoscience & Nanotechnology	196	19,44
Materials Science, Multidisciplinary	184	18,25
Chemistry, Multidisciplinary	151	14,98
Materials Science, Biomaterials	132	13,09
Physical Chemistry	114	11,31
Biochemistry & Molecular Biology	91	9,02
Biomedical Engineering	91	9,02
Surgery	83	8,23
Physics, Applied	71	7,04
Dermatology	69	6,84

Note: A single article may be indexed simultaneously in more than one Web of Science category; therefore, percentages are not mutually exclusive

**Table 2:** Top 10 journals with the highest number of publications

Journal	Number of articles	% of Total
Photodiagnosis and Photodynamic Therapy	62	6,15
Chemical Engineering Journal	47	4,66
ACS Applied Materials & Interfaces	33	3,27
Advanced Healthcare Materials	27	2,67
International Journal of Biological Macromolecules	26	2,57
Lasers in Medical Science	20	1,98
Lasers in Surgery and Medicine	20	1,98
ACS Applied Nano Materials	19	1,88
Journal of Materials Chemistry B	18	1,78
Small	18	1,78

Taken together, these findings indicate that the evolution of the field is no longer driven primarily by improvements in conventional photodynamic therapy protocols but increasingly depends on the integration of photomedicine, materials engineering, and nanotechnology. This convergence has enabled the development of multifunctional therapeutic platforms capable of combining antimicrobial activity, controlled delivery of bioactive agents, and stimulation of tissue repair. This transformation is consistent with trends observed in recent studies, in which smart hydrogels, multifunctional nanoplatforms, metal–organic frameworks (MOFs), and wound microenvironment-responsive systems have assumed a central role in the technological advancement of photodynamic therapy, simultaneously enhancing infection control and the regenerative potential of these therapeutic approaches [30–32].

**Fig. 2:**
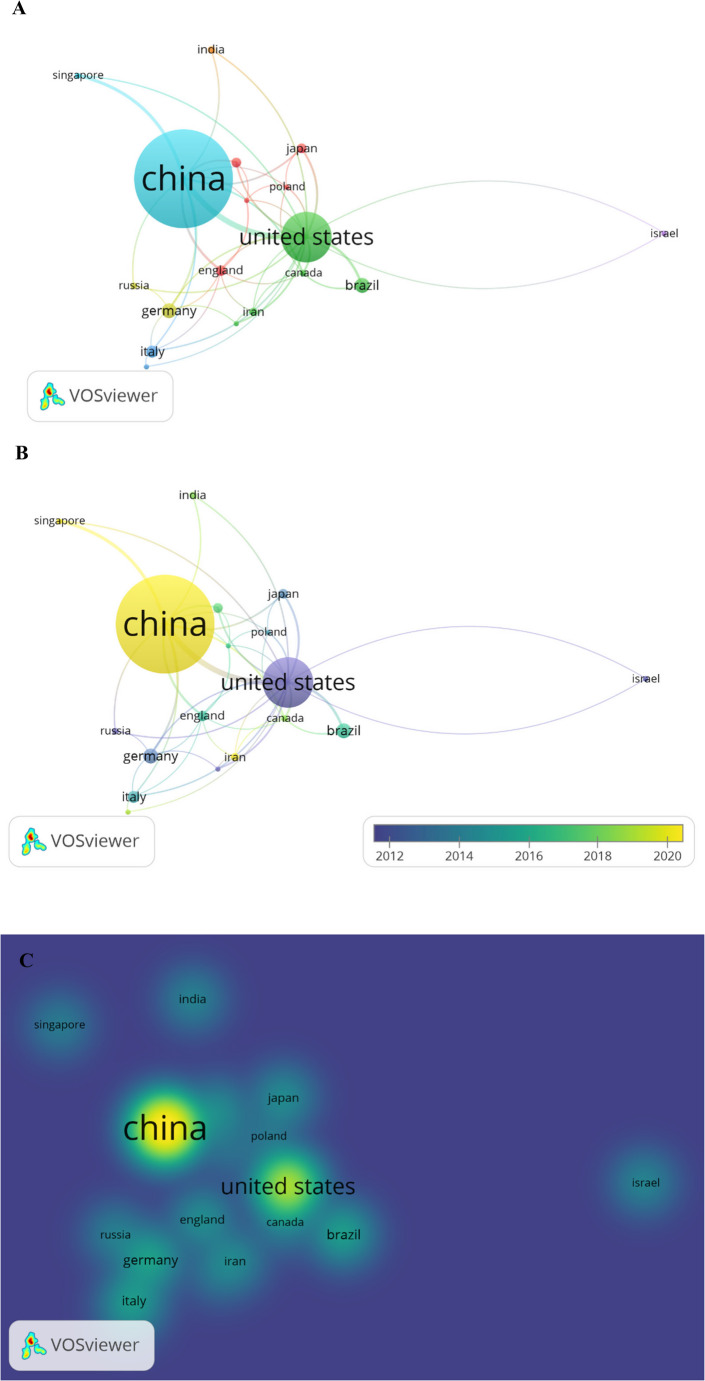
Co-authorship networks among countries. (**A**) Network visualization; (**B**) Overlay visualization; (**C**) Density visualization. Node size represents the number of publications, line thickness represents the strength of connections among countries, and colors indicate clusters or temporal intensity, according to the type of visualization. Source: Prepared by the authors based on analyses performed in VOSviewer

The geographical distribution of scientific production also reveals important changes in the organization of international research. China accounted for more than half of the publications included in the bibliometric corpus, followed by the United States and Brazil (Table 3), whereas the co-authorship analysis identified China and the United States as the principal hubs of international scientific collaboration (Fig. 2A), with China’s leadership becoming increasingly prominent in recent years (Fig. 2B). The density visualization further confirmed these countries as the major global centers of scientific production (Fig. 2C). This prominent role is consistent with the high scientific output observed in the corpus, particularly in studies focusing on nanotechnology, biomaterials, and multifunctional therapeutic platforms.

**Table 3:** Top 10 countries with the highest scientific output among the retrieved articles

Country	Number of articles	% of Total
China	533	52,88
United States	161	15,97
Brazil	61	6,05
Germany	47	4,66
India	34	3,37
Italy	30	2,97
South Korea	26	2,57
England	25	2,48
Russia	25	2,48
Iran	22	2,18

In addition to factors related to scientific capacity, the substantial global burden of cutaneous wounds is a major driver of knowledge production in this field. These lesions affect millions of people worldwide, are highly prevalent among older adults and individuals with chronic conditions such as diabetes mellitus and vascular insufficiency, and are associated with a considerable economic and social burden due to prolonged treatment, recurrence, and infectious complications [7, 33, 34]. In this context, the growing demand for therapeutic strategies capable of controlling infection, promoting tissue repair, and reducing healthcare costs has driven the development of new technologies based on photodynamic therapy, biomaterials, and nanotechnology [32, 35].

The presence of Brazil among the leading contributors also deserves attention. Although its scientific output remains lower than that of China and the United States, this finding may reflect the combination of a high demand for cost-effective therapeutic strategies for the management of chronic wounds and the consolidation of research groups dedicated to the development of technologies for tissue repair. In public healthcare systems, where chronic wounds represent a major clinical and economic challenge, interventions capable of combining microbial control, enhanced wound healing, and cost-effective implementation tend to attract greater scientific interest [36]. In this context, photodynamic therapy possesses characteristics that support its investigation as an alternative approach to improve therapeutic efficacy and optimize healthcare resource utilization [37].

The analysis of the most productive authors indicates that the development of the field has been driven by a relatively small number of researchers, particularly Michael R. Hamblin, Lei Wang, and Yingying Zhang (Table 4). The continuity of their scientific output over the past two decades suggests the consolidation of research lines that have played a significant role in expanding the literature and establishing specialized research groups in photodynamic therapy for cutaneous wound healing. The scientific influence of these researchers is further supported by the historiographic analysis presented later, in which their publications are identified among the foundational works underlying the intellectual development of the field.

**Table 4:** Top 10 authors with the highest number of publications among the retrieved articles

Author	Number of articles	% of Total
Hamblin MR	27	2,67
Wang L	25	2,48
Zhang Y	24	2,38
Liu Y	20	1,98
Wang J	18	1,78
Li Y	14	1,38
Wang X	14	1,38
Dai TH	13	1,29
Wang Y	13	1,29
Zhang XY	12	1,19

## Conceptual evolution of the literature

**Fig. 3:**
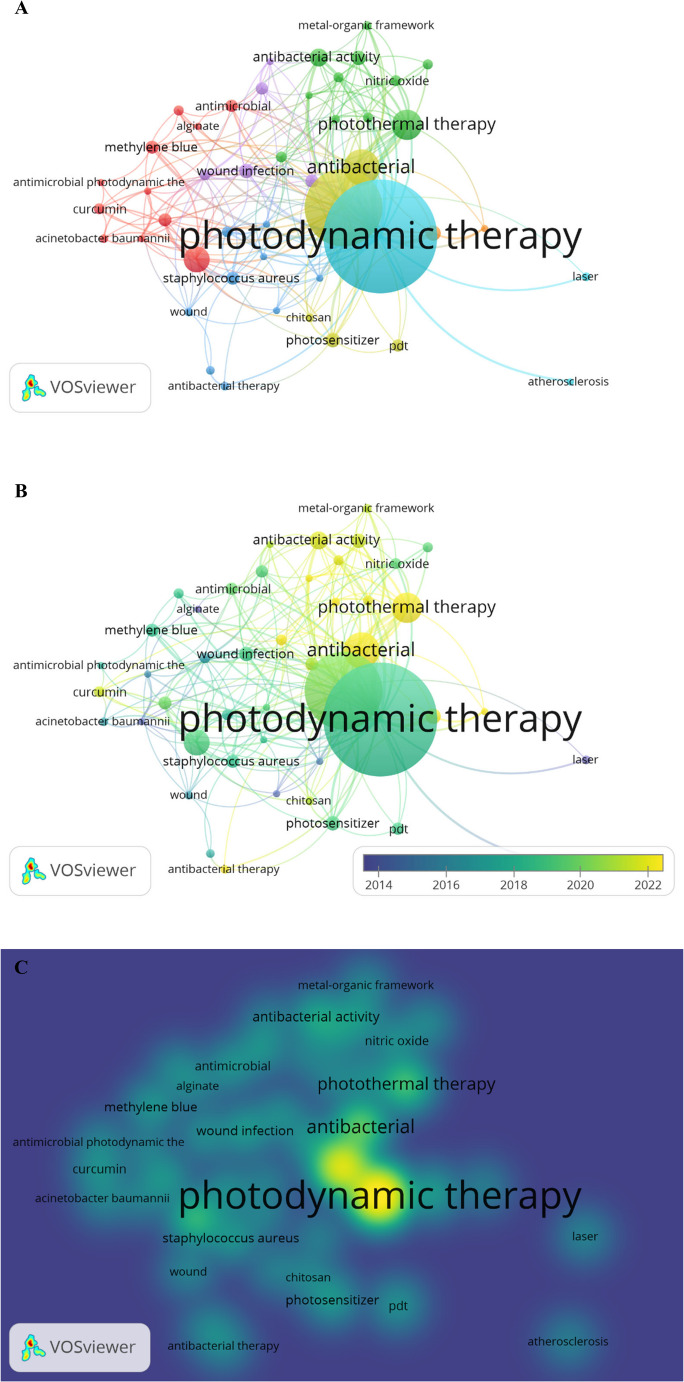
Keyword co-occurrence networks. (**A**) Conceptual network structure; (**B**) Temporal evolution of research topics; (**C**) Term density distribution. Node size represents the frequency of keyword occurrence, whereas lines indicate co-occurrence relationships among terms. Source: Prepared by the authors based on analyses performed in VOSviewer

The keyword co-occurrence analysis revealed a progressive transformation in the conceptual structure of photodynamic therapy for cutaneous wound healing. The co-occurrence network (Fig. 3A) shows that the most central terms in the literature are related to photodynamic therapy, wound healing, infection, antibacterial activity, and reactive oxygen species, reflecting the initial consolidation of the field around the microbiological control of wounds. However, the temporal analysis (Fig. 3B) demonstrates a consistent shift in the thematic profile of the research, characterized by the increasing incorporation of terms related to nanotechnology, biomaterials, hydrogels, biofilms, photothermal therapy, and multifunctional systems. The density visualization (Fig. 3C) further confirms that these topics have become the main focus of the most recent scientific production, indicating a reorganization of the field’s research priorities.

The bibliometric maps indicate a reconfiguration of the conceptual structure of the field. During the early decades, photodynamic therapy was investigated predominantly as a strategy for infection control, driven by the need to develop alternatives to combat the growing challenge of antimicrobial resistance and by experimental evidence demonstrating its effectiveness against wound infections [38–41]. Over time, however, the literature increasingly incorporated biological mechanisms related to inflammation modulation, angiogenesis, fibroblast proliferation, keratinocyte migration, and extracellular matrix remodeling, thereby expanding the role of photodynamic therapy in tissue repair [30, 42]. This evolution resulted from the incorporation of new biological and technological approaches rather than the replacement of the original research lines, leading to a progressive expansion of the therapeutic applications of the technique.

This thematic expansion was accompanied by a marked convergence of photomedicine, nanotechnology, biomaterials science, and regenerative medicine. The emergence of photosensitive hydrogels, nanoparticles, metal–organic frameworks (MOFs), controlled drug delivery systems, and combined strategies involving photodynamic and photothermal therapies demonstrates that research has increasingly focused on multifunctional therapeutic platforms capable of simultaneously targeting infection, inflammation, and tissue regeneration. In addition to overcoming the classical limitations of conventional photodynamic therapy, such as the poor stability of photosensitizers, limited diffusion, and short retention time at the wound site, these technologies have substantially enhanced the translational potential of photodynamic therapy [31, 35, 43]. This conceptual evolution is consistent with the reorganization of the scientific structure observed in the Web of Science categories, the profile of the most productive journals, and the international collaboration networks, demonstrating that the quantitative growth of the literature has been accompanied by qualitative changes in the nature of the research being conducted.

### Intellectual structure of the literature

**Fig. 4:**
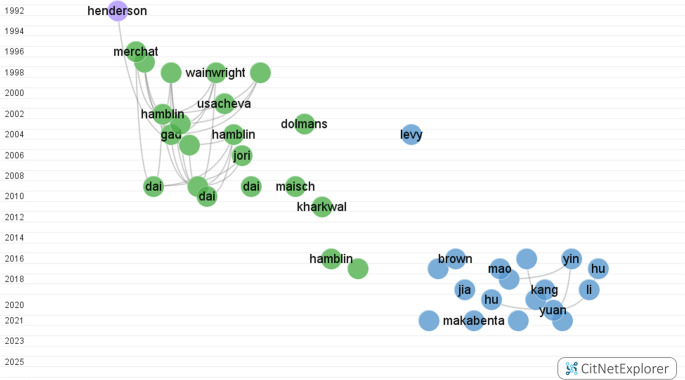
Historiographic citation network of publications on photodynamic therapy for cutaneous wound healing. Nodes represent individual publications, lines represent citation relationships, and colors identify the clusters generated by the CitNetExplorer clustering algorithm (purple = Cluster 1; green = Cluster 2; blue = Cluster 3). Node size is proportional to the Citation Score of each publication. Source: Prepared by the authors based on analyses performed in CitNetExplorer

The historiographic analysis performed using CitNetExplorer enabled the reconstruction of the intellectual trajectory of photodynamic therapy for cutaneous wound healing based on the citation relationships established among the most influential publications in the field (Fig. 4). Unlike the keyword co-occurrence analysis, which highlights the thematic evolution of the literature, the citation network illustrates how knowledge has been progressively accumulated and reorganized over time.

The network structure revealed three clusters automatically identified by the CitNetExplorer clustering algorithm, represented by the different colors in Fig. 4. Cluster 1 (purple) corresponds to a pioneering publication located at the origin of the historiographic network, highlighting its role as the initial reference for the development of the field. Cluster 2 (green) contains most of the publications responsible for the historical structuring of the literature, comprising pioneering and intermediate studies that are strongly interconnected. Cluster 3 (blue) consists predominantly of more recent publications, forming a highly interconnected research core that reflects the contemporary expansion of the field. These clusters represent the structural organization of citation relationships among the analyzed publications and illustrate how different groups of studies have contributed to the consolidation of the literature over time.

However, the interpretation of the intellectual evolution of the field was not based exclusively on the cluster structure. According to the methodological strategy adopted in this study, this interpretation resulted from the integration of the historiographic network, the analysis of publications with the highest centrality and Citation Score, and the findings from the keyword co-occurrence and temporal evolution analyses performed in VOSviewer. This integrated approach made it possible to relate the structural organization of the citation network to the conceptual evolution of the literature, providing a more comprehensive understanding of the scientific development of the field.

Based on this integrated analysis, three major stages in the scientific evolution of photodynamic therapy for cutaneous wound healing were identified. The first stage was characterized by the establishment of the experimental foundations of antimicrobial photodynamic therapy, consolidating concepts related to the use of photosensitizers, the generation of reactive oxygen species, and infection control in wound models [38–41]. Subsequently, this knowledge expanded to include biological mechanisms involved in tissue repair, incorporating investigations on inflammation modulation, angiogenesis, cell proliferation, and the interaction between photodynamic therapy and biomaterials [30]. More recently, the literature has increasingly focused on the development of multifunctional therapeutic platforms integrating nanotechnology, smart biomaterials, wound microenvironment-responsive systems, and combined therapeutic strategies [35, 42, 44]. Collectively, these stages demonstrate that the evolution of the field has occurred in a cumulative manner, with new research directions being incorporated without replacing the foundations established by the pioneering studies.

The convergence between the intellectual structure reconstructed by CitNetExplorer and the conceptual evolution identified through keyword co-occurrence analysis strengthens the interpretation that the expansion of photodynamic therapy for cutaneous wound healing has occurred progressively and in an integrated manner. Whereas the historiographic analysis illustrates how knowledge has accumulated through foundational publications that supported new lines of investigation, the conceptual analysis demonstrates the successive incorporation of new topics, technologies, and applications. Together, these approaches reveal that the growth of the literature reflects not only a quantitative increase in scientific output but also the consolidation of an increasingly interdisciplinary field capable of integrating advances in photomedicine, nanotechnology, biomaterials science, and regenerative medicine to develop more effective therapeutic strategies for the management of cutaneous wounds.

## Limitations and future perspectives

This study has several limitations that should be considered when interpreting its findings. The exclusive use of the Web of Science Core Collection may have excluded publications indexed only in other databases, although this platform is widely recognized for the quality of its bibliographic indexing and the standardization of its metadata. Furthermore, the patterns identified depend on the search strategy and the parameters adopted for constructing the bibliometric networks, whereas publication and citation indicators describe the dynamics of the literature without allowing direct inferences regarding the methodological quality or clinical effectiveness of the included studies. Despite these limitations, the integrated analysis of bibliometric indicators enabled the characterization of the scientific evolution of photodynamic therapy for cutaneous wound healing and the identification of consistent trends in the development of the field. The recent predominance of research involving biomaterials, nanotechnology, and multifunctional therapeutic platforms, together with the need for greater methodological standardization and additional translational studies, highlights promising directions for future research and reinforces the potential of photodynamic therapy as an innovative strategy for the management of cutaneous wounds.

## Conclusions

This bibliometric analysis characterized the scientific evolution of photodynamic therapy for cutaneous wound healing over 25 years of publications indexed in the Web of Science. The findings demonstrated a substantial increase in scientific output, particularly from 2020 onward, accompanied by the consolidation of international collaboration networks, the concentration of scientific production in countries with strong scientific and technological capacity, and the progressive reorganization of the field’s conceptual and intellectual structure. Collectively, the different bibliometric approaches demonstrated that the evolution of the field has occurred in a cumulative manner, integrating knowledge from photomedicine, nanotechnology, biomaterials science, and regenerative medicine.

In addition to providing a comprehensive overview of the scientific literature, this study identified emerging trends, leading researchers and foundational publications, priority research topics, and recent directions in the field, thereby offering valuable insights for planning future investigations and strengthening national and international scientific collaborations. By systematically mapping the organization and evolution of this field of research, this analysis contributes to a broader understanding of the development of photodynamic therapy for cutaneous wound healing and establishes a foundation for guiding future research, fostering the consolidation of innovative technologies, and supporting the advancement of the field toward clinical application.

## Supplementary Information

Below is the link to the electronic supplementary material.

Supplementary Material 1

## Data Availability

The datasets generated and/or analyzed during the current study are available from the corresponding author on reasonable request.
